# The statistical analysis of multi-environment data: modeling genotype-by-environment interaction and its genetic basis

**DOI:** 10.3389/fphys.2013.00044

**Published:** 2013-03-12

**Authors:** Marcos Malosetti, Jean-Marcel Ribaut, Fred A. van Eeuwijk

**Affiliations:** ^1^Biometris - Applied Statistics, Department of Plant Science, Wageningen UniversityWageningen, Netherlands; ^2^Consultative Group on International Agricultural Research Generation Challenge ProgrammeMéxico DF, Mexico

**Keywords:** adaptation, genotype by environment interaction, multi-environment trials, QTL by environment interaction, QTL mapping methodology, REML

## Abstract

Genotype-by-environment interaction (GEI) is an important phenomenon in plant breeding. This paper presents a series of models for describing, exploring, understanding, and predicting GEI. All models depart from a two-way table of genotype by environment means. First, a series of descriptive and explorative models/approaches are presented: Finlay–Wilkinson model, AMMI model, GGE biplot. All of these approaches have in common that they merely try to group genotypes and environments and do not use other information than the two-way table of means. Next, factorial regression is introduced as an approach to explicitly introduce genotypic and environmental covariates for describing and explaining GEI. Finally, QTL modeling is presented as a natural extension of factorial regression, where marker information is translated into genetic predictors. Tests for regression coefficients corresponding to these genetic predictors are tests for main effect QTL expression and QTL by environment interaction (QEI). QTL models for which QEI depends on environmental covariables form an interesting model class for predicting GEI for new genotypes and new environments. For realistic modeling of genotypic differences across multiple environments, sophisticated mixed models are necessary to allow for heterogeneity of genetic variances and correlations across environments. The use and interpretation of all models is illustrated by an example data set from the CIMMYT maize breeding program, containing environments differing in drought and nitrogen stress. To help readers to carry out the statistical analyses, GenStat® programs, 15th Edition and Discovery® version, are presented as “Appendix.”

## Introduction: phenotype, genotype, and environment

The success of a plant breeding program depends on its ability to provide farmers with genotypes with guaranteed superior performance (phenotype) in terms of yield and/or quality across a range of environmental conditions. To achieve this aim, it is necessary to have an understanding of the factors leading to a good phenotype.

Usually the phenotype is the value for a trait at the end of the growing season. The reason is that we are primarily interested in phenotypes like yield or grain weight at maturity and not, or less, in yield or grain weight at earlier stages. The final state of a trait is the cumulative result of a number of causal interactions between the genetic make-up of the plant (the genotype) and the conditions in which that plant developed (the environment). Plants differ in the efficiency and adequacy with which they capture and convert environmental inputs and stimuli into the biomass and organs that constitute a final product. The capture and conversion abilities of a plant are determined by its particular ensemble of genes. Environments differ in the amount and quality of inputs and stimuli that they convey to plants including, e.g., the amount of water, nutrients or incoming radiation. A primary objective in plant breeding is to match genotypes and environments in such a way that improved phenotypes are obtained. For example, a breeder might be interested in selecting genotypes that do well under water stress conditions.

While there can be genotypes that do well across a wide range of conditions (widely adapted genotypes), there are also genotypes that do relatively better than others exclusively under a restricted set of conditions (specifically adapted genotypes). Specific adaptation of genotypes is closely related to the phenomenon of genotype-by-environment interaction (GEI). GEI exists whenever the relative phenotypic performance of genotypes depends on the environment, or in other words, when the difference in reactions of genotypes varies in dependence on the environment.

To illustrate the phenomenon of GEI, we can consider two different genotypes that differ in the genetic machinery involved in tolerance to water-limited conditions, while being equal for all other characteristics. If these two genotypes are exposed to a poorly watered environment, their performance will differ depending on the genetic properties related to tolerance for water-limited conditions. However, this genotypic difference will disappear in an environment that provides the right amount of water. So, the difference in performance between the two genotypes depends on the environment, through the amount of water that it provides.

**Figure 1:**
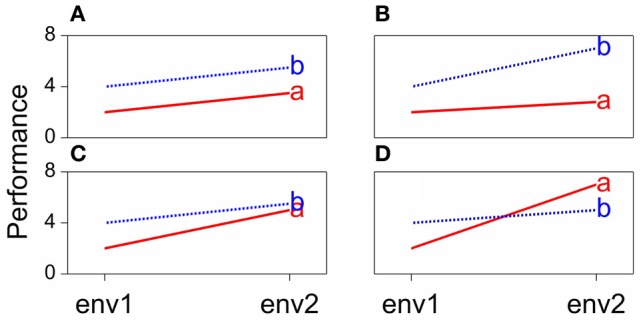
**Genotype-by-environment interaction in terms of changing mean performances across environments: (A) additive model, (B) divergence, (C) convergence, (D) cross-over interaction**.

Some scenarios that can occur when comparing the performances of pairs of genotypes across environments are presented in Figure 1. The function describing the phenotypic performance of a genotype in relation to an environmental characterization is called the “norm of reaction” (Griffiths et al., 1996). Figure 1A shows the case where there is no GEI, the genotype and the environment behave additively (this will be developed later) and the reaction norms are parallel. The remaining plots show different situations in which GEI occurs: divergence (Figure 1B), convergence (Figure 1C), and the most critical one, crossover interaction (Figure 1D). Crossover interactions are the most important for breeders as they imply that the choice of the best genotype is determined by the environment.

**Figure 2:**
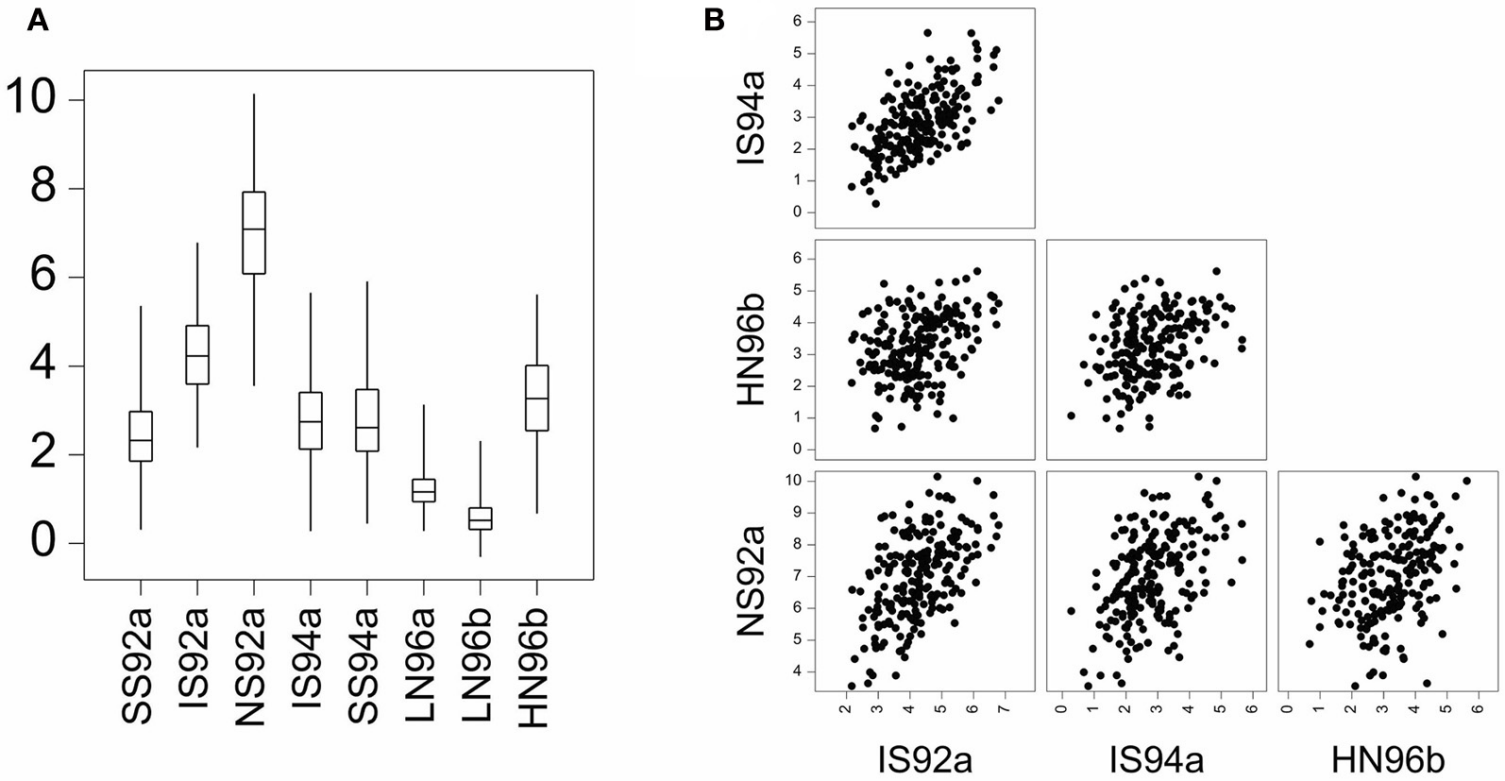
**(A)** Boxplot for yield of a maize F2 population in eight environments displaying total range, interquartile range (box) and median (line). Environment names are coded as: LN, low nitrogen; HN, high Nitrogen; SS, severe water stress; IS, intermediate water stress; NS, no water stress. The two digits indicate the year of the trial, and the letters a and b the cropping season: a, winter; b, summer. **(B)** Scatter plot matrix for two stress environments (IS92a, and IS94a) and two non-stress environments (HN96b and NS92a).

GEI was introduced in terms of the relative difference between genotypic means. GEI can also be regarded in terms of heterogeneity of genetic variance and covariance, or correlation. As a consequence of GEI, the magnitude of the genetic variance as observed within individual environments will change from one environment to the next. Often, the genetic variance tends to be larger in better environments than in poorer environments, although the opposite can be observed as well (Przystalski et al., 2008). Figure 2A illustrates the phenomenon of heterogeneity of genetic variance across environments, showing box plots for a series of maize trials, where the range of variation in the poor environments LN96a and LN96b is smaller than that in the good environments HN96b and NS92a.

GEI has also consequences for the correlations between genotypic performances in different environments. When GEI is large, the observed performance of a set of genotypes in one environment may not be very informative for the performance of the same genotypes in another environment. Environments with similar characteristics will induce corresponding responses in plants and will lead to strong genetic correlations. Figure 2B shows that the correlation between the similar environments IS92a and IS94a is larger than the correlation between the dissimilar environments NS92a and HN96b.

In conclusion, given the complexity of the mechanisms and processes underlying the phenotypic response across diverse and changing environmental conditions—frequently in an unpredictable way—it is necessary to develop analytical tools to help breeders understand GEI. The use of adequate strategies to analyze GEI is a first and important step toward more informed breeding decisions. Good analytical methods are a prerequisite for predicting the performance of genotypes as accurately as possible. This paper explores several strategies to model GEI, starting with simple methods that have been historically popular within the plant breeding community. It then moves to more elaborate models in which additional information is used in the form of explicit environmental characterization to model GEI. A final section is devoted to the integration of molecular marker information into GEI models, leading to the detection of quantitative trait loci (QTLs) and more specifically, to the modeling of QTL by environment interaction (QEI). The statistical methodology is illustrated using a maize data set obtained from a series of drought and nitrogen stress trials from the maize breeding program at Centro Internacional de Mejoramiento de Maíz y Trigo (CIMMYT; the International Maize and Wheat Improvement Center; Ribaut et al., 1996, 1997). To encourage readers to carry out these statistical analyses themselves, GenStat® programs for the 15th Edition (VSN International, 2012) and the Discovery® version of this statistical package (Payne et al., 2007) are presented as “Appendix.”

## Generating data to study genotype-by-environment interaction

An obvious first step to investigate GEI is to obtain phenotypic observations on a set of genotypes exposed to a range of environmental conditions. The set of genotypes can include advanced lines of a breeding program, cultivars, and segregating offspring from a specific cross such as F_2_, a backcross, or a recombinant inbred line (RIL) population.

Genotypes can be tested under different management regimes that represent increasing levels of a particular stress, or a combination of stresses. This type of experiment is called a “managed stress trial” and is appropriate when the researcher wishes to focus on a particular type of stress. When performing managed stress trials, it is important to control the system in such a way that all other factors influencing the phenotype are as homogenous as possible.

Managed stress trials are not a default option in plant breeding, because stress type and level can be difficult to implement and because the relationship between phenotype and stress is complex, with genes and environmental stress(es) interacting throughout the various developmental phases. In those situations, a common way for plant breeders to screen for genotypic reactions to environmental factors is by “multi-environment trials” (METs). In a MET, a number of genotypes are evaluated at a number of geographical locations for a number of years in the hope that the pattern of stresses that the genotypes experience is representative of future growing environments.

A convenient way to summarize data from managed stress trials and METs is in the form of two-way tables of means, with genotypes in the rows and environments in the columns. Each cell of such a table contains an estimate of the performance (adjusted mean) of a particular genotype in a specific environment. To identify genotypes and environments unequivocally, we use indices, the letter *i* for genotypes (*i* = 1… I), and the letter *j* for environments (*j* = 1… J).

The models in the following sections will assume as a starting point a genotype-by-environment table of means. These models are used in a so-called two-stage strategy for analyzing MET data. In the first stage, individual trials are analyzed with models including terms for design features and spatial variation. From these individual trial analyses, adjusted means and weights, usually reciprocals of the variances of the means, are carried forward to the second stage, where a model is fitted to the genotype by environment means, using either no weights or weights estimated in the first stage. Various choices can be made for the weights in a two stage analysis (Mohring and Piepho, 2009; Welham et al., 2010), and a good choice of weights will lead to a two-stage analysis with results very close to those of a so-called single stage analysis, in which plot data are analyzed instead of means. Single stage analyses have certain theoretical advantages over two-stage analyses, but two-stage analyses are logistically and computationally easier to handle. This paper focuses on two-stage analyses, because of the small differences with single stage analyses and the aforementioned larger handling ease. Still, good descriptions of single stage analyses are offered by Cullis et al. (1996a,b), Gilmour et al. (1997), and Smith et al. (2005). In principle, the QTL mapping approach outlined later in this paper could also be embedded in a single stage analysis strategy.

## CIMMYT maize drought stress trials: example data

The models to be presented here are illustrated using data produced by the maize drought stress breeding program of CIMMYT. A brief description of the data is given here, a more detailed description is available in the original publications (Ribaut et al., 1996, 1997). A maize F_2_ population was generated by crossing a drought tolerant parent (P_1_) with a drought susceptible one (P_2_). Seeds harvested from each of 211 F_2_ plants formed F_3_ families, which were stored for further evaluation. The F_3_ families were evaluated in managed stress trials in 1992, 1994, and 1996. In the winter of 1992, a managed water stress trial was conducted in Mexico, including no stress (NS), intermediate stress (IS), and severe stress (SS). In the winter of 1994, a similar trial was conducted, but it only included the IS and SS treatments. In the summer of 1996, the families were tested in a nitrogen stress trial with two levels: low (LN) and high nitrogen (HN). An extra LN trial was conducted in the winter of the same year. In total, the families were evaluated in eight different environments, each environment characterized by year, stress type and intensity, and management factors. DNA was extracted from each of the 211 F_2_ plants to produce a total of 132 restriction fragment length polymorphism (RFLP) markers covering the 10 maize chromosomes.

## Models for genotype-by-environment interaction: modeling the mean

### The additive model as a benchmark

The phenomenon of GEI is of primary interest in plant breeding, and has resulted in a large body of literature on models and strategies for analysis of GEI [see, for example, the reviews in Cooper and Hammer (1996), Kang and Gauch (1996), van Eeuwijk et al. (1996), van Eeuwijk (2006)]. A dominant feature of strategies used to describe and understand GEI is a heavy reliance on parameters that are statistical rather than biological. This is no coincidence, since historically, a large part of quantitative genetics has relied on simple, yet very useful, statistical models. A notorious example is the well-known model: P = G + E, where P stands for phenotype, G for genotype and E for environment (Falconer and Mackay, 1996; Lynch and Walsh, 1998). A statistical formulation of this model for a two-way table of means can be written as:

(1) $${\underline{\mu }}_{ij}=\mu +{\text{G}}_{i}+{\text{E}}_{j}+{\underline{\varepsilon }}_{ij}.$$

From here onwards, in the model formulations, random terms are underlined to emphasize the fact that their effects are assumed to follow a normal distribution. Model 1 describes the response variable, that is, the mean of genotype *i* in environment *j*, μ_*ij*_, as the result of the common fixed intercept term μ, a fixed genotypic main effect corresponding to genotype *i*, G_*i*_, plus a fixed environmental main effect corresponding to environment *j*, E_*j*_, and finally the random term, ε_*ij*_, representing the error term, typically assumed normally distributed, with a mean of zero and constant variance, σ^2^; ε_*ij*_ ~ N(0, σ^2^).

Model 1 predicts that for any genotype the difference means between any two environments *j* and j^*^ will be equal to the difference in the environmental main effects: E_*j*_–E_j^*^_. Consequently, the norms of reaction of genotypes will be parallel (Figure 1A). Another important aspect is that, although the parameters in the model suggest that something intrinsically genetic and something intrinsically environmental is determining the trait, the genotypic and environmental effects purely follow from a convenient way of partitioning phenotypic variation from a statistical point of view. In a balanced data set, the genotypic main effects can be estimated from the average performance of the genotypes across environments. Rather than being something inherently genotypic, this is dependent on the set of environments used in the experiment. If a few environments are dropped, the genotypic effects will change. The same argument applies to the environmental main effects, which depend on the set of genotypes used in the experiment.

The results of the fit of an additive model to the maize data set are presented in Table 1. The results show that, according to the *F*-test, there is a significant environmental and genotypic main effect (the *F* statistic for environments equals 1466.5, and for genotypes 5.3, both of which are highly significant: *P* < 0.001). As just mentioned, environments are characterized by the average performance of the genotypes in the particular environment, and the results indicate that the environments differ significantly in their quality. In general, differences between environmental main effects are significant, and from the breeder's point of view, this is not a major concern. Breeders want to concentrate on differences between genotypes. A significant genotypic main effect indicates that genotypes differ in their average performance across environments, something certainly more interesting to breeders. Finally, it should be mentioned that the residual ε in Table 1 corresponds to the discrepancy between the predicted genotype-by-environment means from an additive model and the observed means.

**Table 1:** **ANOVA table for the additive model (model 1), as applied to CIMMYT maize stress trials**.

**Term**	**Degrees of freedom**	**Sum of squares**	**Mean squares**	***F***	**Probability**
E	7	5679	811.2	1466.5	<0.001
G	210	614	2.9	5.3	<0.001
ε	1470	813	0.6		
Total	1687	7106	4.2		

There are two reasons for the disagreement between the predicted values from an additive model and the observed means for environment-specific genotypic performances: (1) an effect proper to the particular combination of genotype and environment; and (2) experimental error. Model 1 can be extended with an effect that is specific for genotype-by-environment combinations, GEI, or a double-indexed term GEI_*ij*_:

(2) $${\underline{\mu }}_{ij}=\mu +{\text{G}}_{i}+{\text{E}}_{j}+{\text{GEI}}_{ij}+{\underline{\varepsilon }}_{ij}$$

When we are working on a two-way table of means, we cannot straightforwardly separate GEI from error. For that, we would need to develop a model based on plot observations. Use of model 2 implies estimation of as many parameters as there are genotype-by-environment combinations, something that is not desirable in the interest of parsimony. Another limitation of the model is that it is not possible to estimate the genotypic performance in environments that are not included in the trial. Accordingly, fitting model 2 could tell us something about the amount of variation due to genotypic main effects in relation to GEI, by comparing sums of squares or mean squares, but it does not bring much progress toward understanding GEI.

### The regression on the mean model

A more attractive alternative is to extend the additive model (model 1) by incorporating terms that explain as much as possible of the GEI. A popular strategy in plant breeding is that proposed by Finlay and Wilkinson (1963), which describes GEI as a regression line on the environmental quality. In the absence of explicit environmental information, the biological quality of an environment can be reflected in the average performance of all genotypes in that environment. Good environments will have a high average genotypic performance, and bad environments will have a low average genotypic performance. The GEI part is then described by genotype-specific regression slopes on the environmental quality, and the model can be written in the following equivalent ways:

(3a) $${\underline{\mu }}_{ij}=\mu +{\text{G}}_{i}+{\text{E}}_{j}+{\text{b}}_{i}{\text{E}}_{j}+{\underline{\varepsilon }}_{ij}$$

(3b) $${\underline{\mu }}_{ij}={\text{G}}_{i}^{\prime }+{\text{b}}_{i}^{\prime }{\text{E}}_{j}+{\underline{\varepsilon }}_{ij}$$

Model 3b follows from model 3a by taking μ + G_*i*_ = G′_*i*_ and E_*j*_ + b_*i*_E_*j*_ = (1 + b_*i*_)E_*j*_ = b′_*i*_E_*j*_. Model 3b is easier to interpret because it looks as a set of regression lines; each genotype has a linear reaction norm with intercept G′_*i*_ and slope b′_*i*_. The explanatory environmental variable in these reaction norms is simply the environmental main effect E_*j*_. Model 3a shows more clearly how GEI is captured by a regression on the environmental main effect, with the hope that as much as possible of the GEI signal will be retained by the term b_*i*_E_*j*_.

In the regression on the mean model, GEI is explained in terms of differential sensitivities to the improvement of the environment, with some genotypes (the ones with larger values of b_*i*_) benefiting more than others from an increase in environmental quality. Note that in model 3a, Σb_*i*_ = 0, so that the average slope value is zero, while in model 3b the average value of b′ is 1, meaning that b′ > 1 for genotypes with a higher than average sensitivity, and b′ < 1 for genotypes that are less sensitive than average.

Table 2 gives the fit of model 3a to the maize example data. The first two rows of the table, corresponding to the genotypic and environmental main effects, are identical to Table 1. The third row corresponds to the GEI effect in terms of the regression on environmental quality, where quality is represented by the environmental mean. This regression is highly significant, according to the *F*-tests (*F* = 2.4, *P* < 0.001). The residual sum of squares in Table 1 (SS_ε_ = 813) has been divided into a part explained by genotypic sensitivities to environmental quality (SS_b_ = 230), and a residual (SS_ε_ = 583).

**Table 2:** **ANOVA table for the regression on the mean model (model 3), as applied to CIMMYT maize stress trials**.

**Term**	**Degrees of freedom**	**Sum of squares**	**Mean squares**	***F***	**Probability**
E	7	5679	811.2	1752.3	<0.001
G	210	614	2.9	6.3	<0.001
Heterogeneity of slopes	210	230	1.1	2.4	<0.001
ε	1260	583	0.5		
Total	1687	7106	4.2		

**Figure 3:**
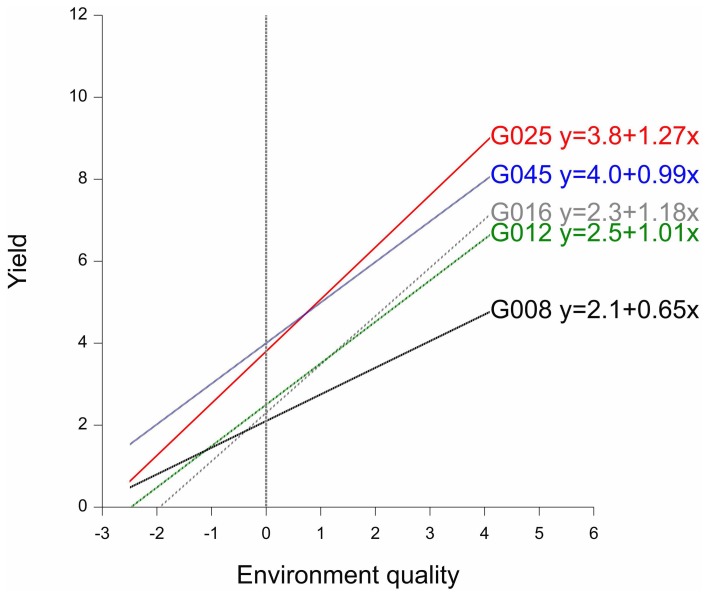
**Finlay–Wilkinson regression curves of five maize genotypes.** The vertical line indicates the average environment. Next to genotype labels, the corresponding Finlay-Wilkinson regression equation is given.

By way of example, the fitted reaction norms of five genotypes (out of the full set of 211 genotypes) are given in Figure 3, together with the parameters estimated according to the parameterization in model 3b (G′ and b′). Figure 3 shows that, in the average environment, genotypes G025 and G045 are better than G008, G012, and G016. The estimates for the parameters G′ can be read-off from the plot as the fitted values at the null value of the *x*-axis, i.e., the average environment indicated by the dashed vertical line. Although G045 does slightly better than G025 in the average environment, G025 is superior to G045 in the high-quality environments. This is because G025 has a better ability to exploit improved environmental conditions, which is reflected in its higher genotypic sensitivity (b′_G025_ = 1.27 > b′_G045_ = 0.99). A similar observation can be made for G008 vs. G012 and G016. While G008 does relatively better in low quality environments, it is clearly surpassed by G012 and G016 in the best environments, since it is not capable of profiting from the better environmental conditions (b′_G008_ = 0.65, which is the lowest sensitivity among the five genotypes).

In summary, the regression on the mean model describes GEI in terms of parameters that can be given some biological meaning. In addition, and in contrast with the full interaction model (model 2), model 3 can be used to predict the performance of genotypes in environments that were not present in the MET, as long as the environment for which predictions are required can reasonably be placed within the range of environments used in the original MET. Nevertheless, the regression on the mean model suffers from the fact that the environmental characterization is based on a single dimension. Environmental quality can be hard to summarize within a single explanatory variable. Therefore, a substantial amount of GEI can remain unexplained. In the next section, the regression on the mean model will be extended by including multidimensional environmental characterizations in the statistical model for the genotype-by-environment data.

### The additive main effects and multiplicative interactions model

The limitation of a single dimension in environmental characterization can be removed by employing a more flexible model, in which more than one environmental quality variable is allowed. A popular model of this type is the additive main effects and multiplicative interaction (AMMI) model (Gollob, 1968; Mandel, 1969; Gabriel, 1978; Gauch, 1988; van Eeuwijk, 1995). To emphasize the similarities with model 3a, we write the AMMI model as:

(4) $${\underline{\mu }}_{ij}=\mu +{\text{G}}_{i}+{\text{E}}_{j}+\sum_{k=1}^{\text{K}}{\text{b}}_{ik}{\text{z}}_{jk}+{\underline{\varepsilon }}_{ij}$$

where the GEI is now explained by K multiplicative terms (*k* = 1… K), each multiplicative term formed by the product of a genotypic sensitivity b_*ik*_ (genotypic score) and a hypothetical environmental characterization z_*jk*_ (environmental score). Although genotypic and environmental scores are deemed to represent genetic and environmental qualities, they come from a mathematical procedure, a principal components analysis on the GEI (Gabriel, 1978; Gauch, 1988) that maximizes the variation explained by the products of the genotypic and environmental scores. The first product term is the one that explains most of the variation, followed by the second one, and so on. This is reflected in Table 3, which shows the results from the AMMI model to the maize example data. In the AMMI model, GEI is explained by two axes (principal component 1, PCA1, and principal component 2, PCA2) that are highly significant (*F* = 2.8 and 2.0 respectively, both with an associated *P* < 0.001). The first axis (PCA1) explains the largest part (SS_PCA1_ = 242), the second one explains a little less (SS_PCA2_ = 173), with a total explained sum of squares for GEI of 242 + 173 = 415, an improvement over the explained sum of squares in the regression on the mean model (SS_b_ = 230).

**Table 3:** **ANOVA table corresponding to application of AMMI2 model (model 4) to CIMMYT maize stress trials**.

**Term**	**Degrees of freedom**	**Sum of squares**	**Mean squares**	***F***	**Probability**
E	7	5679	811.2	1752.3	<0.001
G	210	614	2.9	6.3	<0.001
PCA1	216	242	1.1	2.8	<0.001
PCA2	214	173	0.8	2.0	<0.001
ε	1040	398	0.4		
Total	1687	7106	4.2		

PCA1 and PCA2 are the principal component axes 1 and 2, respectively.

**Figure 4:**
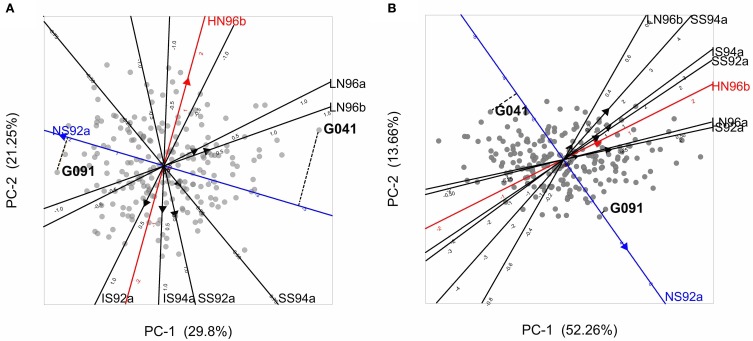
**(A)** Biplot from the AMMI model used to describe GEI in the maize example data. Gray circles represent genotypes, and filled triangles environments, with triangles pointing in the direction of increasing GEI (at origin GEI = 0). The projection of two genotypes (G041 and G091) on the NS92a axis is shown by a dashed line. **(B)** GGE biplot for the maize data set, with same characteristics as of the AMMI biplot, except that triangles point in the direction of increasing overall performance (G + GEI), so the origin corresponds to the average performance of all genotypes in the particular environment. Projections for genotypes G041 and G091 are given.

A desirable property of the AMMI model is that the genotypic and environmental scores can be used to construct powerful graphical representations called biplots (Gabriel, 1978) that help to interpret the GEI. Figure 4A presents a biplot for the maize data. A first thing to recognize is that both genotypes and environments are present in the same plot; genotypes are represented by gray circles and environments by filled triangles (red, blue, and black). The environments are typically represented as axes intersecting at their origins. The origins represent the averages for the trait in the corresponding environments. The triangles point in the direction of increasing trait values. By projecting genotypes on environmental axes, GEI for individual genotypes is approximated. To help interpretation, environmental axes can be enriched by including a scale (Graffelman and van Eeuwijk, 2005).

Biplots facilitate the exploration of relationships between genotypes and/or environments. Genotypes that are more similar to each other are closer to each other in the plot than genotypes that are less similar. The same is true for environments. Genotypes/environments that are alike tend to cluster together. The angle between environmental axes is related to the correlation between the environments. An acute angle indicates positive correlation (e.g., between LN96a and LN96b), a right angle indicates no correlation (e.g., between HN96b and NS92a), and an obtuse angle indicates negative correlation (e.g., NS92a and LN96a). The projection of a genotype onto an environmental axis reflects the performance of that genotype in that environment (for GEI). For example, genotype G091 projects on the NS92a axis above the origin, indicating a positive interaction with that environment i.e., the relative performance (GEI part) of G091 in NS92a is above the average of all genotypes in NS92a. Conversely, genotype G041 (on the right hand side of the plot) projects below the origin on the same axis, which points to a negative interaction with environment NS92a (i.e., G041 performs worse than average). Following a similar procedure it is possible to conclude that while genotype G091 showed positive adaptation to environment NS92a, it is not well adapted to environments LN96a and LN96b (the projection of G091 on the LN96a and LN96b axes falls below the origin). Biplots are useful tools to investigate patterns in GEI, because they can help to identify interesting genotypes that are adapted to particular environments, and to classify environments in groups.

Plant breeders are interested in the total genetic variation and not exclusively in the GEI part. For that reason, it is useful to have a modification of model 4 that considers the joint effects of the genotypic main effect and the GEI as a sum of multiplicative terms. Effectively, the two-way table of genotype-by-environment means is exposed to a standard principal components analysis, with genotypes as objects and environments as variables (Yan et al., 2000). For this model, closely the same estimation and interpretation procedures hold as for model 4. Because genotypic scores now describe genotypic main effects G and GEI together, this type of model is also known as the “Genotype main effects and GEI model,” or “GGE model” and the biplots are called “GGE biplots” (Yan et al., 2000). The model reads:

(5) $${\underline{\mu }}_{ij}=\mu +{\text{E}}_{j}+\sum_{k=1}^{\text{K}}{\text{b}}_{ik}{\text{z}}_{jk}+{\underline{\varepsilon }}_{ij}$$

The results of model 5 fitted to the maize data are presented in the form of a biplot in Figure 4B. GGE biplots approximate overall performance (G + GEI). This is in contrast to AMMI biplots, Figure 4A, that approximate only the GEI part of the phenotype. Figure 4B shows the high yielding genotypes concentrated on the right hand side of the biplot, with their projections on environmental axes covering the above average range (for example, G091 projects above the origin in NS92a, whereas G041 is found below the origin). In contrast, low yielding genotypes (as G041) are concentrated on the left hand side of the biplot (projects below origin in most of the environments).

### Factorial regression models

The models discussed so far assumed that we do not have explicit information about the environments. While such models can be useful to explain GEI, the biological interpretation of their results is not always obvious. What do hypothetical environmental variables, as in AMMI, mean in terms of quantifiable environmental characteristics such as temperature, water, nutrients etc? A straightforward approach is to correlate environmental scores with environmental covariables. However, if we do have explicit information about the environment, the information can be used directly in the model by including it in the form of explanatory variables. GEI is then described as differential genotypic sensitivity to explicit environmental factors such as temperature, precipitation, water availability etc. Such models are known as factorial regression models (Denis, 1988; van Eeuwijk et al., 1996). Two examples of factorial regression models are given here. Model 6a includes a single environmental covariable, while model 6b includes multiple environmental covariables:

(6a) $${\underline{\mu }}_{ij}=\mu +{\text{G}}_{i}+{\text{E}}_{j}+{\text{b}}_{i}{\text{Z}}_{j}+{\underline{\varepsilon }}_{ij}$$

(6b) $${\underline{\mu }}_{ij}=\mu +{\text{G}}_{i}+{\text{E}}_{j}+\sum_{k=1}^{\text{K}}{\text{b}}_{ik}{\text{Z}}_{jk}+{\underline{\varepsilon }}_{ij}$$

Models 6a and 6b look very similar to models 3a and 4, but there is a substantial difference between them. In models 6a and 6b, Z_*j*_ represents an explicit environmental covariable and not a hypothetical environmental covariable as in models 3a and 4 (note that Z is capitalized to highlight this difference). This distinction is critical since the interpretation of the GEI in models 6a and 6b is automatically placed into a biological context. Instead of describing GEI as differential reactions to hypothetical environmental covariables, factorial regression models help to identify genotypes that are differentially sensitive to changes in identified environmental quality components, for example, in a particular nutrient, or in water availability.

Table 4 shows the results of a factorial regression model fitted to the maize example data, in which GEI is explained by differential genotypic sensitivities to the minimum temperature during flowering (minTF, *F* = 1.7, *P* < 0.001) and to the amount of radiation during grain filling (radiationGF, *F* = 1.2, *P* ≤ 0.038). In many cases, different combinations of explanatory variables could produce closely similar models in terms of the amount of explained GEI. Therefore, to arrive at biologically meaningful models, it is crucial to combine statistical criteria for model selection with physiological knowledge about the trait that is involved (Voltas et al., 1999a,b, 2002).

**Table 4:** **ANOVA table corresponding to application of a factorial regression model (model 6) to CIMMYT maize stress trials**.

**Term**	**Degrees of freedom**	**Sum of squares**	**Mean squares**	***F***	**Probability**
E	7	5679	811.2	1752.3	<0.001
G	210	614	2.9	6.3	<0.001
G.minTF	210	172	0.8	1.7	<0.001
G.radiationGF	210	124	0.6	1.2	≤0.038
ε	1050	517	0.5		
Total	1687	7106	4.2		

## Mixed models for genotype-by-environment interaction: modeling genetic variances and covariances

In the introduction, it was mentioned that GEI can be regarded both in terms of differential mean responses across environments and in terms of heterogeneity of genetic variation and covariation between environments. While the models considered so far focus on modeling the mean response, the models in this section focus on the modeling of GEI in terms of heterogeneity of variances and covariances. This section switches to the framework of so-called mixed models. We concentrate on the main characteristics of a few, relatively simple yet powerful, mixed models that can be used to model GEI in terms of heterogeneity of variance and covariance. A more detailed description of mixed models can be found in the literature elsewhere (Verbeke and Molenberghs, 2000; Galwey, 2006).

The models discussed in the previous sections were all examples of fixed effects models, with all terms except the residual term fixed. However, genotypes can be regarded as a random sample from a larger population (especially easy when the number of genotypes is large, say more than 10), in which case genotypes are an extra source of random variation. This situation calls for a mixed model, with genotypes taken as random term. A review of the use of mixed models to analyse complex data sets in plant breeding can be found in Smith et al. (2005). For the maize example data set, there are 211 genotypes. When the genotypic main effects are taken as random, the following mixed model equivalent of the additive model can be defined as:

(7) $${\underline{\mu }}_{ij}=\mu +{\underline{\text{G}}}_{i}+{\text{E}}_{j}+{\underline{\varepsilon }}_{ij}$$

$${\underline{\text{G}}}_{i}\sim \text{N}(0,\sigma_{\text{G}}^{\text{2}})\;{\underline{\varepsilon }}_{ij}\sim \text{N}(0,\sigma_{\varepsilon }^{\text{2}})$$

The term G_*i*_ is underlined to indicate that it is a random term; its distribution needs to be specified, and usually is taken to be normal, with zero mean and a variance specific to the term. Model 7 contains two variance components, one corresponds to the random genotypic main effects, σ^2^_G_, and a second one, σ^2^_ε_, corresponds to the residual (which includes true GEI and error). An important consequence of including genotypes as random is that automatically genetic covariances and correlations between performances in different environments are imposed. The total variance for individual genotypic observations in a particular environment *j*, σ^2^_*j*_, is the sum of two sources of variation: σ^2^_*j*_ = σ^2^_G_ + σ^2^_ε_. The covariance between observations for a particular genotype in environments *j* and j^*^, σ_jj^*^_, following from model 7 is: σ_jj^*^_ = σ^2^_G_. For observations on different genotypes σ_jj^*^_ = 0. In model 7, similarities (or covariation, and therefore correlation) between observations made on the same genotype in different environments are assumed to be positive, but covariation between observations on different genotypes (regardless whether the observation is done in the same or in different environments) is assumed to be zero. Model 7 is referred as the compound symmetry model (Verbeke and Molenberghs, 2000).

The general definition for a correlation between two traits, or two environments, *x* and *y* is:

$$r_{\left(x;\;y\right)}=\frac{\text{covariance}(x;y)}{\sqrt{\text{var}(x)}\sqrt{\text{var}(y)}}$$

Model 7 imposes a constant correlation between environments, with the correlation between any pair of environments *j* and j^*^ (for clarity, we write Env_*j*_ and Env_j^*^_ when referring to those environments), being equal to:

$$r_{\left({\text{Env}}_{j};\;{\text{Env}}_{j^{*}}\right)}=\frac{\sigma_{jj^{*}}}{\sqrt{\sigma_{j}^{2}}\sqrt{\sigma_{j^{*}}^{2}}}=\frac{\sigma_{\text{G}}^{2}}{\sqrt{\sigma_{\text{G}}^{2}+\sigma_{\varepsilon }^{2}}\sqrt{\sigma_{\text{G}}^{2}+\sigma_{\varepsilon }^{2}}}=\frac{\sigma_{\text{G}}^{2}}{\sigma_{\text{G}}^{2}+\sigma_{\varepsilon }^{2}}$$

Although mixed models can be fitted by standard least squares procedures in the case of balanced data, a more general method of inference to fit mixed models is by residual maximum likelihood, or REML (Patterson and Thompson, 1971). Results of analyses based on REML are presented in another way than the familiar ANOVA tables. Table 5 shows the results obtained by fitting mixed models to the maize example data.

**Table 5:** **REML output of the fit of different mixed models to the CIMMYT maize stress trials**.

**Model 7**			**Model 8**			**Model 9**		
**Fixed**	**Wald (*DF*)**	***P***	**Fixed**	**Wald (*DF*)**	***P***	**Fixed**	**Wald (*DF*)**	***P***
*E*	10265.3 (7)	<0.001	*E*	9759.4 (7)	<0.001	*E*	6268.8 (7)	<0.001
**Random**	**Estimate**	***SE***	**Random**	**Estimate**	***SE***	**Random**	**Estimate**	***SE***
σ^2^_G_	0.297	0.036	σ^2^_G_	0.125	0.017	σ^2^_C1_	0.439	0.053
σ^2^_ε_	0.553	0.020	σ^2^_ε1_	0.551	0.057	σ^2^_C2_	1	–
			σ^2^_ε2_	0.692	0.071	σ^2^_C3_	0.042	0.013
			σ^2^_ε3_	1.399	0.140	σ_C1C2_	0.551	0.077
			σ^2^_ε4_	0.672	0.069	σ_C1C3_	0.109	0.019
			σ^2^_ε5_	0.704	0.072	σ_C2C3_	0.115	0.032
			σ^2^_ε6_	0.135	0.018	σ^2^_ε1_	0.446	0.051
			σ^2^_ε7_	0.152	0.019	σ^2^_ε2_	0.445	0.052
			σ^2^_ε8_	0.761	0.078	σ^2^_ε3_	0.736	0.169
						σ^2^_ε4_	0.428	0.050
						σ^2^_ε5_	0.508	0.057
						σ^2^_ε6_	0.145	0.018
						σ^2^_ε7_	0.138	0.017
						σ^2^_ε8_	0.740	0.080
**Deviance (*DF*)**	**1077.9 (1678)**		**Deviance (*DF*)**	**838.4 (1671)**		**Deviance (*DF*)**	**619.9 (1667)**	

*Model 7 assumes compound symmetry, model 8: assumes heterogeneity of genetic variance across environments, and model 9 assumes heterogeneity of genetic covariance between groups of environments and heterogeneity of genetic variance across individual environments. Environments are indexed as: 1 = SS92a, 2 = IS92a, 3 = NS92a, 4 = IS94a, 5 = SS94a, 6 = LN96a, 7 = LN96b, 8 = HN96b. Groups of environments are indexed as: C1 = SS92a, IS92a, IS94a, SS94a, HN96b; C2 = NS92a; C3 = LN96a, LN96b*.

Table 5 does not contain sums of squares, nor mean squares. Instead, there is a table with three main sections. For model 7, the compound symmetry model, one section contains the results for testing fixed model terms (header Fixed terms). A second section shows the estimates for the variances of the random terms (header Random terms), and a third section a goodness-of-fit statistic, the deviance, that can be used to compare mixed models with equal fixed terms and differing random terms (header Deviance). For the fixed effects (environments in this case), Table 5 shows a Wald test statistic, the corresponding degrees of freedom (DF), and a *P* value. The Wald test statistic is used to assess the significance of fixed effects in the REML mixed model framework. Under the null hypothesis of no fixed effects, the Wald test has a distribution that is approximately a Chi-square with DF equal to the number of independent effects for the particular fixed term. In the maize example, the Wald test statistic for environments is 10,265.3 and it has 8−1 = 7 degrees of freedom. This Wald statistic has a very low tail probability in the Chi-square distribution under the null hypothesis of no environmental effects (*P* < 0.001). So, it is concluded that there is a significant difference between environments. Some statistical packages, including GenStat®, can provide an *F*-distributed approximation to the Wald statistic.

The estimates of the two parameters associated to the random terms in the model: σ^2^_G_ = 0.297 and σ^2^_ε_ = 0.553 are given in the second part of Table 5. The magnitude of the variance components can be compared to have an impression of the relative importance of genotypic main effects (σ^2^_G_) in relation to the sum of GEI and error (σ^2^_ε_). The genetic correlation between any two environments is estimated as:

$$r_{\left({\text{Env}}_{j};\;{\text{Env}}_{j^{*}}\right)}=\frac{0.297}{0.297+0.553}=0.349$$

The last row in Table 5 presents the deviance (equal to −2 times the restricted loglikelihood), which is a measure of how well the model fitted to the data. The better the model, the lower the deviance is. As will be seen later, the deviance can be used to compare different models to select the best model for the data, provided that the fixed part of the model remains unchanged.

Model 7 assumes a constant genetic variance and correlation between pairs of environments. For METs, the assumption of constant genetic variance and genetic correlation across environments is unrealistic (Figure 2A). In the presence of GEI, a more realistic model would allow the total genetic variance to change from environment to environment, which will in turn, cause heterogeneous genetic correlations between environments:

(8) $${\underline{\mu }}_{ij}=\mu +{\underline{\text{G}}}_{i}+{\text{E}}_{j}+{\underline{\varepsilon }}_{ij}$$

$${\underline{\text{G}}}_{i}\sim \text{N}(0,\sigma_{\text{G}}^{2})\;{\underline{\varepsilon }}_{ij}\sim \text{N}(0,\sigma_{\varepsilon_{j}}^{2})$$

In model 8, there is still a single genetic variance component for genotypes, and therefore, a constant genetic covariance between environments. However, the variance for the term ε_*ij*_ that includes GEI and error, is assumed to depend on the environment (i.e., the variance component σ^2^_ε*j*_ is indexed by *j*). Table 5 presents the results of fitting model 8 to the maize data. Instead of two variance components, there are now nine, one corresponding to the variance component for genotypes (σ^2^_*G*_ = 0.125), and eight corresponding to a form of GEI for each of the eight environments (for convenience, we assume constant errors). The heterogeneity of variance for ε_*ij*_ reflects that in some environments there is a larger variation (e.g., in environment 3, which is the high-yielding NS92a) than in other environments (e.g., in environments 6 and 7, which are low-yielding, LN96a and LN96b). The heterogeneity of variance leads to heterogeneous genetic correlations between environments. For example, the correlation between environments 6 and 7 is:

$$r_{\left({\text{Env}}_{6};\;{\text{Env}}_{7}\right)}=\frac{0.125}{\sqrt{0.125+0.135}\sqrt{0.125+0.152}}=0.466$$

and between environments 3 and 6 is:

$$r_{\left({\text{Env}}_{3};\;{\text{Env}}_{6}\right)}=\frac{0.125}{\sqrt{0.125+1.399}\sqrt{0.125+0.135}}=0.199$$

In conclusion, model 8 accommodates heterogeneity of variance between environments and, with it, allows for heterogeneous correlations between environments, which can be desirable when analyzing environments that strongly differ (e.g., with strong stress and without stress).

The deviance for model 8 is 838.4 with 1671 DF, which is much lower than the one for model 7 (deviance 1077.9 with 1678 DF). The deviance has dropped, but at the expense of having to estimate more parameters (nine instead of two parameters). Is the decrease in deviance large enough to consider model 8 a significant improvement over model 7? Because model 7 and 8 are nested models (model 7 is a special case of model 8 when the σ^2^_ε*j*_ are equal for all *j*), a deviance test can be used to answer this question. Under the null hypothesis of no difference in quality of the fits, the difference in deviance between the two models is Chi-square distributed with the number of DF equal to the difference in the number of parameters between the models. In the example, the difference in deviance is 1077.9 − 838.4 = 239.5, and the models differ by seven parameters. The P value associated to 239.5 in a Chi-square distribution with 7 DF is very small (*P* < 0.001), so it is concluded that model 8 provides a significant improvement over model 7.

In cases where the models are not nested, the comparison can be done by the Akaike Information Criterion (AIC) (Akaike, 1974). For model 7, AIC = 4170, and for model 8 AIC = 3944. The model that has the lowest AIC value is the one that is chosen. Model 8 has the lowest AIC value, which agrees with the conclusion based on the deviance test.

Model 8 assumes heterogeneous variances across environments, in combination with a constant covariance between environments. This latter assumption can be relaxed by also allowing the genetic covariance between environments to be heterogeneous. A possibility is to estimate a covariance parameter for each pair of environments, producing a variance-covariance model that is referred to as the “unstructured model” (Verbeke and Molenberghs, 2000). A somewhat simpler strategy consists of estimating covariances between groups of environments instead of between individual environments, in which the environments are first grouped in a number of clusters and then fitting the following model:

(9) $${\underline{\mu }}_{i(\text{c})j}=\mu +{\underline{\text{G}}}_{i(\text{c})}+{\text{E}}_{j}+{\underline{\varepsilon }}_{i(\text{c})j}$$

$${\underline{\text{G}}}_{i(\text{c})}\sim \text{N}(0,\Sigma_{\text{c}})\;{\underline{\varepsilon }}_{i(\text{c})j}\sim \text{N}(0,\sigma_{\varepsilon_{j}}^{2})$$

In model 9 a random genetic main effect is fitted that changes between groups of environments and that has a covariance matrix Σ_*c*_ that consists of group specific genetic variances, with σ^2^_*cj*_ for group *j*, on the diagonals, and pairwise-specific genetic covariances, with σ_cjj^*^_between groups *j* and j^*^, on the off-diagonals. Model 9 retains the residual heterogeneity of model 8, which means that environment specific genotypic effects are added to group specific genotypic effects. To illustrate model 9, using the maize example, and based on Figure 4, the environments were clustered in three groups: group 1 = (SS92a, SS94a, IS92a, IS94a, HN96b), group 2 = (NS92a), and group 3 = (LN96a, LN96b). Therefore, the covariance matrix Σ_C_ will contain on the diagonal the genetic variances for groups 1, 2, and 3 (σ^2^_c1_, σ^2^_c2_, and σ^2^_c3_ respectively), and on the off-diagonals the covariances between the groups (σ_c12_, σ_c13_, and σ_c23_). The full covariance matrix can be written as:

$$\Sigma_{\text{C}}=\left(\begin{array}{ccc} \sigma_{\text{c1}}^{\text{2}} & & \\ \sigma_{\text{c12}} & \sigma_{\text{c2}}^{\text{2}} & \\ \sigma_{\text{c13}} & \sigma_{\text{c23}} & \sigma_{\text{c3}}^{\text{2}} \end{array}\right)$$

The results of fitting model 9 to the maize data are presented in Table 5, where the estimates of the parameters in the covariance matrix Σ_C_ can be found.

The diagonals of Σ_C_ show that, on average, the genetic variation is lower in group 1 (the group of nitrogen stress environments) than in group 2. It should be noted that because group 3 is composed of a single environment, the genetic variation cannot be partitioned into a component due to the group and a residual, so σ^2^_c3_ is not estimated but arbitrarily fixed to 1. The total variance in each of the environments is equal to the sum of the group's variance plus the environment-specific variance. For example, the variance in environment 1 is equal to 0.885, which is the sum of the variance of group 1, i.e., σ^2^_c1_ = 0.439, and σ^2^_ε1_ = 0.446. Recalling that the covariance between environments within the same group is given by σ^2^_c1_, σ^2^_c2_ and σ^2^_c3_, and the covariance between environments in different groups by σ_c1c2_, σ_c1c3_, and σ_c2c3_, the correlation between any pair of environments can be estimated. For example, the correlation between environments 1 and 2 is:

$$r_{\left({\text{Env}}_{1};\;{\text{Env}}_{2}\right)}=\frac{0.439}{\sqrt{0.439+0.446}\sqrt{0.439+0.445}}=0.496$$

and between environments 1 and 7 is:

$$r_{\left({\text{Env}}_{1};\;{\text{Env}}_{7}\right)}=\frac{0.109}{\sqrt{0.439+0.446}\sqrt{0.042+0.138}}=0.273$$

Finally, the deviance can be used to evaluate whether the allowance for heterogeneity of covariance between environments improved the quality of the model or not.

The deviance for model 9 is 619.9 with 1667 DF, and the difference in deviance with model 8 is 218.5, with four extra parameters. The associated *P* value for 218.5 in a Chi-square distribution with 4 DF is very low (*P* < 0.001), so it can be concluded that model 9 is a significant improvement over model 8. For model 9 AIC = 3736, which is smaller than for model 8 (AIC = 3944), and confirms this conclusion.

We have presented different mixed model formulations to model GEI in terms of heterogeneity of variance and covariance between environments. The compound symmetry model, which is the commonly used default model when fitting a mixed model to a two–way table of means, forces variances and covariances to be constant across environments. Two alternative models accommodated either heterogeneity of genetic variances across environments, or heterogeneity of genetic variances and covariances across environments. There are other useful variance-covariance models such as the factor analytic (Malosetti et al., 2004; Boer et al., 2007) that combines flexibility with parsimony (reduced number of parameters), but their discussion is outside the scope of this paper.

The analysis of a data set is an iterative process consisting of fitting and comparing alternative models to identify a good model for the data under study. That process has been illustrated with a maize data set. The next section goes one step further in the modeling process by including molecular marker information, with the ultimate objective of identifying genomic regions, QTLs, that underlie genetic variation of quantitative traits. Within the context of METs, the use of such models is a powerful tool to identify and understand the genetic basis of GEI, that is, QEI.

## QTL mapping in the context of multi-environment trials: modeling main effect QTLs and QTL-by-environment interaction

So far, we discussed models that use either implicit or explicit environmental characterizations to understand GEI. We switch in this section to the use of explicit *genotypic* information in the models describing GEI. Use of such information in statistical models for GEI can help understand the basis of GEI in terms of the action of genome regions, QTLs, in their dependence on the environment, i.e., QEI. Molecular marker systems (RFLP, AFLP, DArT, SSR, SNP) provide information about variation at the DNA level that can be employed in statistical models. For example, within the framework of factorial regression models, markers can serve as explanatory variables, which is at the core of regression–based approaches for QTL mapping (Haley and Knott, 1992; Martínez and Curnow, 1992).

Elaborating upon factorial regression ideas, the following section presents mixed models that can accommodate explicit genotypic information to describe GEI in terms of QTL and QEI effects (Malosetti et al., 2004; Boer et al., 2007; van Eeuwijk et al., 2007, 2010). The genotypic information stemming from markers is introduced in the statistical models in the form of so-called genetic predictors. Applications of mixed model QTL by environment detection as the one described here, can be found in wheat (Mathews et al., 2008), sugar cane (Pastina et al., 2012), and sorghum (Sabadin et al., 2012). We should emphasize, that although we focus on QTL models applied to standard biparental populations, these models can be adapted rather easily to multi-parental populations (van Eeuwijk et al., 2010; Huang et al., 2011), or association mapping panels (Malosetti et al., 2007; van Eeuwijk et al., 2010).

While here we focus in this paper on mixed model QTL detection, this is certainly not the only method for multi-environment QTL mapping. A well known and common alternative is to use mixture model approaches (Jiang and Zeng, 1995), for which various user-friendly QTL software packages exist (e.g., QTL Cartographer, Basten et al., 2002). However, such QTL software packages typically provide little or no opportunity to intervene with the statistical model, nor do they allow for applying different model building strategies. For example, in the mixture model context, it is hard to switch between different models for representing the dependencies between environments or add explicit information on the environments, something that is relatively easy in the mixed model context.

## Explanatory variables for differences between genotypes: genetic predictors

Most populations in QTL mapping originate from crosses between pairs of inbred lines. A segregating offspring population can be produced from an F_1_ hybrid after one generation of selfing (F_2_), after several generations of self-pollination (recombinant inbred lines or RIL), or after crossing the F_1_ with one of the parental lines (backcross). In addition, by chromosome doubling of F_1_ gametes, a population of doubled haploid lines can be generated. In all of these cases, two alleles at most will segregate at each locus. For a locus *M_1_*, individuals can have the genotypes M_1_M_1_, M_1_m_1_, or m_1_m_1_, with M_1_ the allele that comes from the paternal line, and m_1_ the allele that comes from the maternal line. By convention the locus names are given in italics (so for example *M_1_* refers to locus 1, and M_1_ and m_1_ refer to the paternal and maternal alleles at locus 1, respectively). The relative frequency of the genotypes in the offspring population depend on the type of population; for example, in an F_2_ the expected frequencies are ¼, ½, and ¼ for M_1_M_1_, M_1_m_1_, and m_1_m_1_, respectively.

With the help of molecular markers, it can be revealed whether a particular individual is of the M_1_M_1_, M_1_m_1_, or m_1_m_1_ type. To detect QTLs and estimate their effects, it is necessary to translate the marker information into explanatory variables or genetic predictors. A straightforward way of constructing genetic predictors is to create an explanatory variable that contains the number of copies of one of the alleles, for example, the M_1_ allele. The genetic predictor will then take the value 2 whenever an individual has two paternal alleles (M_1_M_1_), the value 1 when the offspring individual is M_1_m_1_, and 0 when it is m_1_m_1_. Using a simple regression model, the slope for the regression of the genotypic means on a genetic predictor defined by the number of M_1_ alleles corresponds to the effect of a substitution of an m_1_ allele by an M_1_ allele at the given locus (Lynch and Walsh, 1998; Bernardo, 2002). This effect is also known as the additive genetic substitution effect of the QTL allele. By analogy, a dominance genetic predictor can be constructed by creating an explanatory variable with values 0, when the offspring individual is M_1_M_1_ or m_1_m_1_, and value 1 whenever it is M_1_m_1_.

With complete information on the marker genotypes, i.e., codominant markers without missing values, the construction of genetic predictors at marker positions consists of simply counting the number of alleles coming from a particular parent. For genomic positions in between marker loci (putative QTL positions), for dominant markers, and for markers with missing values, the construction of genetic predictors requires more effort. In a general formulation, the value for the additive genetic predictor, X^add^, for an offspring individual can be defined as the expected number of alleles coming from the paternal line, the number of M_1_ alleles:

(10a) $$\begin{array}{l} {\text{X}}^{\text{add}}=\text{Pr}({\text{M}}_{1}{\text{M}}_{1}|\text{all markers})\times 2+\text{Pr}({\text{M}}_{1}{\text{m}}_{1}|\text{all markers})\\ \;\times 1+\text{Pr}({\text{m}}_{1}{\text{m}}_{1}|\text{all markers})\times 0, \end{array}$$

with Pr(M_1_M_1_|all markers), Pr(M_1_m_1_|all markers), and Pr(m_1_m_1_|all markers) the conditional probabilities of the individual being of the M_1_M_1_, M_1_m_1_, or m_1_m_1_ type, respectively given the observed marker information. Note that in the case of complete information, the individual's genotype is known, so one of Pr(M_1_M_1_|markers), Pr(M_1_m_1_|markers) and Pr(m_1_m_1_|markers) will be equal to 1, while the others will be 0.

In the case of incomplete information, although the genotype for a locus of an individual may not be known with certainty, information can be obtained from nearby markers to estimate the probability of the offspring individual being of a particular genotype. This probability is a function of the observed genotypes at neighboring markers and the expected recombination occurring between those marker loci and the locus under evaluation (Lynch and Walsh, 1998). Efficient methods to calculate conditional genetic probabilities for the different types of population commonly used for plants have been proposed in the literature; see Jiang and Zeng (1997) for an exhaustive overview. The calculation of genotypic probabilities conditional on marker information provides the basis for all QTL mapping strategies; QTL mapping packages calculate these probabilities behind the scenes. In GenStat® (see “Appendix”), a very general Hidden Markov Model algorithm has been programmed to calculate those condtional probabilities. Other packages that calculate those probabilities and that are free are *Grafgen* (Servin et al., 2002) and r/qtl (Broman et al., 2003).

With the estimated conditional probabilities, the genetic predictors at positions where no or partial marker information is available can be calculated by using the conditional probabilities in expression 10a. An analogous reasoning holds for the estimation of dominance genetic predictors:

(10b) $$\begin{array}{l} {\text{X}}^{\text{dom}}=\text{Pr}({\text{M}}_{1}{\text{M}}_{1}|\text{all markers})\times 0+\text{Pr}({\text{M}}_{1}{\text{m}}_{1}|\text{all markers})\\ \;\times 1+\text{Pr}({\text{m}}_{1}{\text{m}}_{1}|\text{all markers})\times 0. \end{array}$$

## Modeling genotype-by-environment interaction in terms of QTL effects

The inclusion of genetic predictors in a GEI model allows testing the hypothesis that the DNA at a particular genome position has an effect on a phenotypic trait, and whether that effect is environment dependent or not. A basic GEI phenotypic model, as the one discussed in the previous sections, can be extended to accommodate two new terms, one for the additive genetic effect of a possible QTL (X^add^_*i*_α_*j*_), and a second for the dominance effect of the same locus (X^dom^_*i*_δ_*j*_):

(11) $${\underline{\mu }}_{ij}=\mu +{\text{E}}_{j}+{\text{X}}_{i}^{\text{add}}\alpha_{j}+{\text{X}}_{i}^{\text{dom}}\delta_{j}+{\underline{\text{G}}}_{i}+{\underline{\varepsilon }}_{ij},$$

where X^add^_*i*_, and X^dom^_*i*_ stand for the values of the additive and dominance genetic predictors of individual *i* at the position at which a QTL is postulated and tested for. The parameters α_*j*_ and δ_*j*_ represent the additive and dominance effects of this QTL. In model 11, both types of QTL effects are indexed by *j*, because environment-specific effects are allowed. Residual genetic main effects (i.e., genetic effects not explained by the QTL) contribute to the random genetic effect, G_*i*_, and residual GEI (residual QEI) contributes to ε_*ij*_. The conclusion about the presence of a QTL at a particular position is based on a Wald test (Verbeke and Molenberghs, 2000) that assess the null hypothesis of the environment-specific additive and dominance genetic effects being zero across all environments: Ho: α_*j*_ = 0, and Ho: δ_*j*_ = 0, *j* = 1… J. Note that as by definition, dominance effects are deviations from additivity, so dominance effects should be tested conditional on the additive effects present in the model. In practice, and to assure that the proper test is used, it is adviced to include the term for additive genetic effects in the model before the term for the dominance effects, and use the sequential Wald test (e.g., in GenStat® output, the test under the heading “Sequentially adding terms to fixed model”).

For the maize data, Table 6 shows an example of the application of model 11 to a particular genomic position. The table indicates that the dominance effect at this genome position was not significant (Wald statistic = 13.5 on 8 DF, *P* ≤ 0.097), and, therefore, the null hypothesis of no dominance effects is not rejected. However, the Wald statistic for the additive genetic effects was highly significant (Wald = 100.9, on 8 DF, *P* < 0.001), indicating the existence of additive QTL effects. It is still necessary to find out whether they are environment specific, i.e., whether a QEI term is needed, or whether a model with just main effect QTL expression would suffice. To this purpose, the environment–specific QTL effects (α_*j*_) are partitioned into an additive main effect (α^*Q*^) and QEI effects (α^QEI^_*j*_), leading to the following model:

(12) $${\underline{\mu }}_{ij}=\mu +{\text{E}}_{j}+{\text{X}}_{i}^{\text{add}}\alpha^{\text{Q}}+{\text{X}}_{i}^{\text{add}}\alpha_{j}^{\text{QEI}}+{\text{X}}_{i}^{\text{dom}}\delta_{j}+{\underline{\text{G}}}_{i}+{\underline{\varepsilon }}_{ij}$$

**Table 6:** **Results of the test for fixed effects in a mixed model including a fixed environment–specific additive ( α *_*j*_*) and dominance (δ_*j*_) QTL effect**.

**Fixed terms**	**Wald**	***DF***	***P***
E	10875.5	7	<0.001
Additive effect (α_*j*_)	100.9	8	<0.001
α^*Q*^	12.8	1	<0.001
α^QEI^_*j*_	88	7	<0.001
Dominance effect (δ_*j*_)	13.5	8	≤0.097

*The additive QTL effect is partitioned into a QTL main effect (α^Q^), and a QEI effect (α^QEI^_j_)*.

If required, a similar partitioning of the QTL effects may be carried out for the dominance effects. As a result of the partitioning of the environment-specific QTL effects, there is a Wald test for QTL main effect and a Wald test for QEI (Table 6). The QEI effects should be tested, conditional on the main effect being fitted into the model, i.e., the QTL main effect should always precede the term for QEI. In the example, it is observed that the QEI interaction effect is highly significant (Wald = 88.0 on 7 DF, *P* < 0.001), so it is concluded that QTL effects are dependent on the environment. Since there is significant QEI, no attempt will be made to interpret the QTL main effect. When QEI is not significant, the model can be simplified by omitting the QEI term, as the QTL main effect will suffice to describe the QTL effect.

## A QTL mapping strategy for multi-environment trials based on mixed models

The preceding section presented a number of models that can be useful in the detection of QTLs for MET data. The present section discusses a strategy for a genome-wide scan for QTLs. QTL mapping can be regarded as a model selection process aiming to identify a model that describes the phenotypic response in terms of QTL effects. Since a priori neither the number of QTLs nor their effects are known, we need a strategy that allows to explore the vast range of possible models. There is no unique way of performing this search, but an effective strategy is presented here consisting of the following steps: (1) find a good model for the phenotypic data; (2) perform a genome–wide scan for QTLs by simple interval mapping (SIM); (3) perform one or more rounds of composite interval mapping (CIM) starting with cofactors selected from the SIM step; and (4) fit a final multi–QTL model to estimate QTL effects. Each step is illustrated using the maize example data. An example code that performs the different steps in GenStat® (VSN International, 2012) and in GenStat Discovery® (Payne et al., 2007) is given in the “Appendix.”

### Step 1: identify the best variance-covariance model for the phenotypic data

A number of models can be fitted (for example models 7 to 9 plus the unstructured model), and compared based on the AIC values. The selected mixed model will be the starting point from which to develop a QTL model. Table 7 gives the AIC for four candidate models for the maize example data, and shows that the unstructured model is the best (lowest AIC) and is, therefore, chosen as the basic phenotypic model.

**Table 7:** **Comparison of the goodness of fit for four different mixed models (models 7 to 9 and the unstructured model), as fitted to CIMMYT maize stress trials**.

**Model**	**Deviance**	***DF***	**Δ Deviance**	**Δ *DF***	***P***	**AIC**
Model 7	1077.9	1678	–	–	–	4170
Model 8	838.4	1671	239.5	7	<0.001	3944
Model 9	619.9	1667	218.5	4	<0.001	3736
Unstructured	548.7	1644	71.2	23	<0.001	3708

*The columns “Δ deviance” and “Δ DF” indicate the differences in deviance and number of degrees of freedom between the current and the preceding model in the list. The associated P values correspond to a Chi-square distribution with Δ DF degrees of freedom*.

### Step 2: genome-wide QTL scan, simple interval mapping

After choosing the phenotypic model, a genome-wide scan is performed by fitting single QTL models across the genome at marker and in between marker positions, i.e., SIM. To perform SIM, we need to estimate genetic predictors that cover the genome. For most population types and population sizes of a few hundred individuals, calculating the genetic predictors every 5–10 cM is sufficient. The genetic predictors are used to test for QTL effect at the predictor location. The unstructured model was selected for the maize data set, so the SIM scan can be done by fitting the following model at every genetic predictor position (only additive effects are tested as a previous analysis showed little dominance):

(13) $${\underline{\mu }}_{ij}=\mu +{\text{E}}_{j}+{\text{X}}_{i}^{\text{add}}\alpha_{j}+{\underline{\text{G}}}_{i}+{\underline{\varepsilon }}_{ij}$$

**Figure 5:**
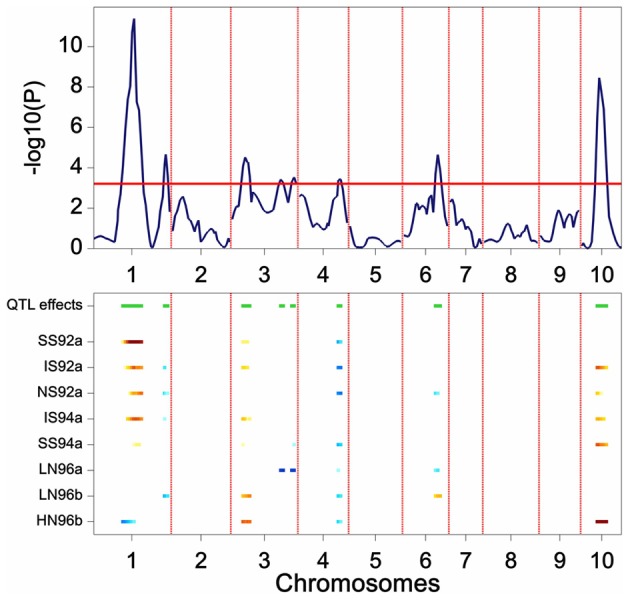
**Plot produced by a SIM QTL scan in a maize F2 population.** The upper panel shows the *P* value of the Wald test (on a –log10 scale) for the effect of a QTL along the chromosomes (solid line). The horizontal line indicates a threshold value for significance. The lower panel gives an indication of magnitude of QTL effects (higher intensity, larger effect), and parental line contributing the superior allele (blue, maternal; red, parental line).

The results of a genome-wide SIM scan are plotted in Figure 5. The upper plot displays the *P* value of the Wald test (on a –log_10_ scale) for the effect of a QTL along the chromosomes. The horizontal line indicates a threshold value, above which the null hypothesis of no QTL is rejected. The profile shows evidence of QTLs on chromosomes 1, 3, 4, 6, and 10. The two largest QTLs are the ones on chromosome 1 and on chromosome 10. The lower panel shows an indication of the magnitude of the QTL effects in each of the environments at a particular chromosome position. The type of color points to the parent that contributes the high value allele (blue = maternal line, red = paternal line), and the color intensity to the magnitude of the effect. QEI is reflected in this plot by changes in color at a particular chromosome position (cross-over interaction) or by changes in intensity of the color (convergence-divergence). For example, the large QTL on chromosome 1 not only shows changes in magnitude of the effects between environments (different color intensities), but also shows change of colors. For example, while in HN96b the allele increasing yield comes from the mother (blue), in IS92a, IS94a, NS92a, SS92a, and SS94a the allele increasing yield comes from the father (red). This is an example of cross-over interaction. The large QTL on chromosome 10 shows only differences in magnitude of the QTL effect (from largest in HN96b to no effect in LN96a, LN96b, and SS92a), but always with the allele from the father contributing to higher yield.

Scanning the results across the full set of chromosomes produces a list of putative QTL positions that can be used as cofactors at the following stage of the QTL mapping.

SIM implies performing multiple tests along the genome, one test at each putative QTL position. For example, for the maize data genetic predictors were calculated at 246 chromosome positions, which means that model 13 was fitted 246 times. When performing multiple tests, the probability of at least one false positive (i.e., falsely rejecting the null hypothesis) increases according to the expression 1 − (1 − α)^*n*^, with α the test level for a single test and n the number of tests. A simple correction method is the Bonferroni correction that uses α/*n* instead of α to test individual null hypotheses, assuring that the proportion of false rejections among *n* tests will be at most equal to α. For example, to accept a maximum of 5% of false rejections in the whole of the experiment (genome–wide), one should use a threshold equal to 0.05/*n*. A disadvantage of the Bonferroni correction is that it is very conservative risking that some QTLs may go undetected, especially when not all tests are independent, which is the case in QTL mapping where nearby positions are correlated.

Modifications to the Bonferroni correction in the context of QTL mapping have been proposed by Cheverud (2001), and further modifications proposed by Li and Ji (2005). Both approaches essentially compensate for the fact that, in QTL mapping, tests are correlated by using an estimated effective number of tests (n^*^) instead of the actual number of tests (*n*) to set the significance threshold. For the maize data, the Li and Ji (2005) approach produced a value of n^*^ = 81, which gives a larger threshold *P* value than the Bonferroni correction (divide 0.05 by 81, instead of dividing by 246). By default, GenStat estimates n^*^ and uses it to set the corresponding significance threshold.

### Step 3: composite interval mapping

The power of QTL detection can be improved by reducing the background noise caused by QTLs outside the region under test. This is the principle of the CIM approach, simultaneously proposed by Jansen and Stam (1994) and Zeng (1994). What makes the difference between SIM and CIM, is that when performing CIM the model includes a number of cofactors that corrects for the effects of the genetic background:

(14) $${\underline{\mu }}_{ij}=\mu +{\text{E}}_{j}+\sum {\text{X}}_{if}{\text{c}}_{jf}+{\text{X}}_{i}^{\text{add}}\alpha_{j}+{\underline{\text{G}}}_{i}+{\underline{\varepsilon }}_{ij}$$

In model 14 the term ∑ X_*if*_c_*jf*_ accounts for the effects of QTLs outside the region that is being tested (X^add^_*i*_), reducing the error variation and thereby improving the power for QTL detection. Various strategies exist for the selection of a set of cofactors, but a pragmatic approach is to use the results from the SIM scan, including the positions indicative of QTLs by SIM as cofactors.

Another issue that needs to be addressed is that when testing in a region close to a cofactor, it is necessary to exclude the particular cofactor from the model to avoid colinearity with the tested position. A popular solution is to choose a window around an evaluation position such that if a cofactor falls inside that window, then the cofactor is excluded from the model. Window size affects the results of a CIM scan, and there are no clear–cut recommendations about which window size to use. For the present example, all cofactors that are on the chromosome being evaluated are excluded, a strategy known as restricted CIM.

**Figure 6:**
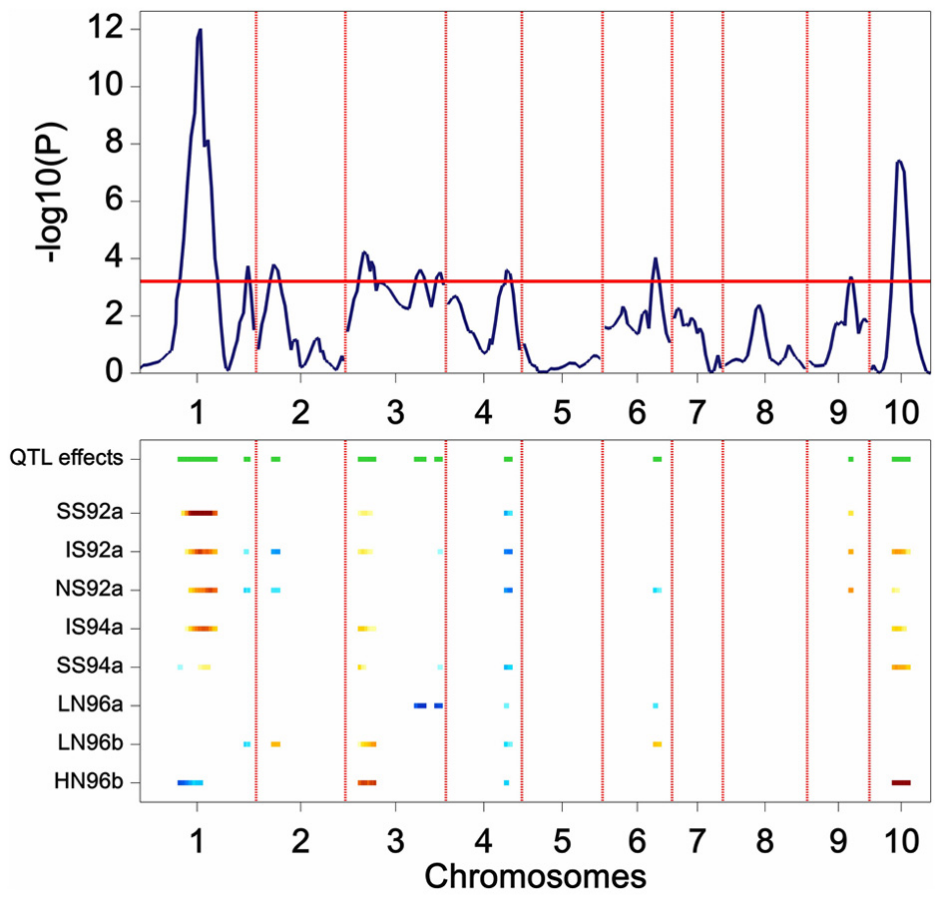
**Plot produced by a CIM QTL scan in a maize F2 population.** The upper panel shows the *P* value of the Wald test (on a –log10 scale) for the effect of a QTL along the chromosomes (solid line). The horizontal line indicates a threshold value for significance. The lower panel gives an indication of magnitude of QTL effects (higher intensity, larger effect), and parental line contributing the superior allele (blue, maternal; red, parental line).

The results of the restricted CIM scan for the maize data are presented in Figure 6. The profiles point to QTLs on chromosomes 1, 2, 3, 4, 6, 9, and 10. In comparison with the results from SIM, the CIM profile reveals the same QTLs (the two major QTLs on chromosome 1 and 10, and the ones on chromosome 3, 4, and 6), but in addition it shows indications of QTLs on chromosomes 2 and 9.

### Step 4: establishing a final QTL model

In a subsequent modeling step, the QTLs for all positions that were found significant in the restricted CIM scan are included simultaneously in the mixed model:

(15) $${\underline{\mu }}_{ij}=\mu +{\text{E}}_{j}+\sum {\text{X}}_{iq}^{\text{add}}\alpha_{jq}+{\underline{\text{G}}}_{i}+{\underline{\varepsilon }}_{ij}$$

Model 15 is a multi–QTL model constructed by inclusion of the full set of QTLs identified in the previous CIM scan. QTLs with non-significant effects will be removed using Wald tests (conditional on all other QTLs) to arrive at a final model. The final model for our example data showed that nine out of the ten QTLs from the CIM scan were significant in the multi-QTL model. Further, by breaking down the QTL effects into QTL main effects (α^*Q*^_*q*_) and QEI effects (α^QEI^_*q*_), it was possible to investigate whether QTL effects were consistent across environments or not. All QTLs but the one at the end of chromosome 3, had significant QEI (*P* < 0.01).

The estimated QTL effects are given in Table 8. The effect of a QTL in a particular environment is declared significant when zero is outside the confidence interval of the estimated effect (CI = estimate ± 2^*^s.e., with s.e. the average standard error obtained from the REML analysis). Results for the large QTL on chromosome 1 (QTL_1,141_) showed that the QTL had a significant effect of 0.469 ton·ha^−1^ in environment SS92a, which means that for each replacement of the maternal allele by a paternal allele, a yield increase of about half a ton is expected. The effect of the same QTL in environment HN96b had a negative sign (−0.232 ton·ha^−1^), which means that rather than an increase, a decrease in yield is expected for the same allele substitution. The effects of QTL_1,141_ are inconsistent across environments not only in terms of the size of the effects, but also in terms of the sign of the effect. Inconsistency in size and sign of QTL effects underlies crossover interactions, the most important case of GEI (recall Figure 1D). From the breeder's point of view, the crossover QEI means that, while the maternal allele has to be selected when breeding for environment HN96b, the paternal allele will be the choice when selecting for all the other environments. The other large QTL, which is on chromosome 10 (QTL_10,67_) showed changes of the sizes of the effects but not of their signs, indicating that the favorable allele came always from the paternal line. The size of the QTL effect was largest in HN96b (0.564 ton·ha^−1^), around 0.300 ton·ha^−1^ in IS92a, IS94a, NS92a, and SS94a, and not significant in LN96a, LN96b, and SS92a. Despite changes in effect sizes, in this case, selection will always be for the paternal allele. In contrast to these two QTLs, the QTL at 217 cM on chromosome 3 (QTL_3,217_) showed a consistent effect across all environments (−0.129 ton·ha^−1^) with the maternal allele as the yield increasing allele. The other QTLs showed different degrees of interaction with the environment, involving crossovers (QTL_2,36_ and QTL_6,125_) or only differences in magnitude of effects (QTL_1,252_, QTL_3,38_, QTL_4,136_, and QTL_9,97_). The QTL effect information is useful at the moment of selecting complementary lines that combine in future crosses the favorable alleles coming from the maternal and paternal line.

**Table 8:** **QTL effect estimates (ton·ha^−1^) for individual environments**.

	**SS92a**	**IS92a**	**NS92a**	**IS94a**	**SS94a**	**LN96a**	**LN96b**	**HN96b**
QTL_1, 141_	0.469^*^	0.351^*^	0.370^*^	0.370^*^	0.214^*^	−0.005	−0.002	−0.232^*^
QTL_1, 252_	−0.026	−0.078	−0.292^*^	−0.061	0.182	−0.05	−0.106^*^	0.093
QTL_2, 36_	−0.123	−0.304^*^	−0.329^*^	−0.026	−0.091	−0.003	0.131^*^	0.106
QTL_3, 38_	0.224^*^	0.236^*^	0.035	0.323^*^	0.241^*^	−0.007	0.152^*^	0.480^*^
QTL_3, 217_	−0.129^*^	−0.129^*^	−0.129^*^	−0.129^*^	−0.129^*^	−0.129^*^	−0.129^*^	−0.129^*^
QTL_4, 136_	−0.272^*^	−0.344^*^	−0.456^*^	−0.147	−0.293^*^	−0.093^*^	−0.107^*^	−0.262^*^
QTL_6, 125_	−0.006	0.015	−0.332^*^	0.061	0.004	−0.096^*^	0.116^*^	−0.155
QTL_9, 97_	0.187^*^	0.251^*^	0.386^*^	0.016	0.023	0.026	−0.018	0.021
QTL_10, 67_	0.056	0.324^*^	0.258^*^	0.251^*^	0.322^*^	0.072	0.054	0.564^*^

*A positive sign indicates that the superior allele comes from the parental line, and a negative sign indicates the superior allele comes from the maternal line. QTL effects significantly different from zero are indicated with an asterisk*.

## Modeling QTL effects in relation to environmental information

An interesting possibility with the QTL models presented here is that they allow the inclusion of environmental information to explain QTL effects in terms of sensitivities to environmental factors. Similarly to GEI models in which environmental information can be integrated to describe GEI effects, QEI models can integrate environmental information to describe QEI effects. Expressing QTL effects in terms of sensitivities to a particular environmental factor allows prediction of the effect of the QTL under any condition within the range of the original experiments. In addition, the inclusion of environmental information can help unravel the physiological mechanisms that are behind the action of a particular QTL.

**Figure 7:**
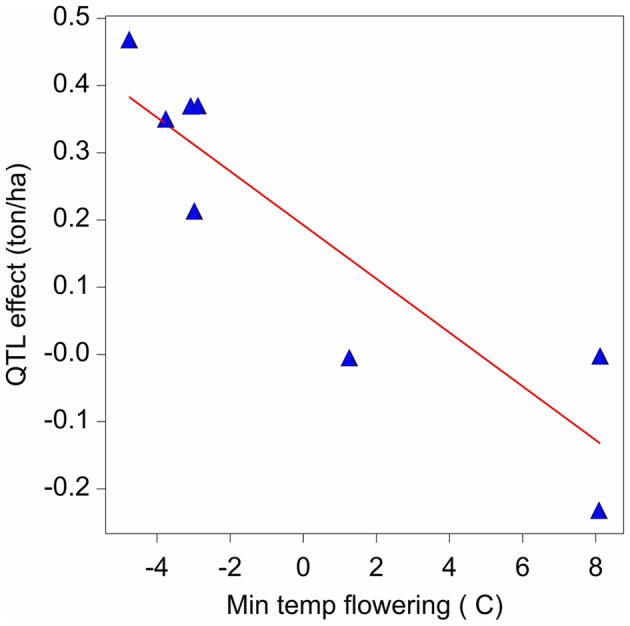
**Effect on yield (ton ha-1) of the QTL on chromosome 1 at 141 cM in relation to the minimum temperature (°C) during flowering time**.

The final QTL model for the maize example data consisted of nine QTLs. It can now be investigated as to whether the variation in effects of those QTLs is related to changes in one or more external environmental variables (There exists a strong analogy with the factorial regression models discussed for GEI, models 6a and 6b). Figure 7 presents a scatter plot of the QTL_1,141_ effects across environments vs. the minimum temperature during flowering time. The plot shows a negative relationship between the QTL effect and temperature.

Assuming a simple linear relationship between the effect of a QTL and a given environmental covariable, it is possible to test for that relationship using the following model:

(16) $${\underline{\mu }}_{ij}=\mu +{\text{E}}_{j}+\sum {\text{X}}_{iq}^{\text{add}}\alpha_{jq}+{\text{X}}_{i}(\alpha_{q^{*}}+\beta_{q^{*}}{\text{Z}}_{j}+{\underline{\text{a}}}_{jq^{*}})+{\text{G}}_{i}+{\underline{\varepsilon }}_{ij}$$

For simplicity, in model 16, the regression of environment-specific QTL effects on environmental covariables is developed for one QTL (q^*^). However, the procedure can be applied equally well to other QTLs with environment–specific effects. In model 16, the effect of the QTL is expressed in relation to an environmental covariable (Z), where the effect of the QTL is equal to: α_jq^*^_ = α_q^*^_ + β_q^*^_Z_*j*_ + a_jq^*^_. Z_*j*_ represents the value of the covariable Z for environment *j*. When Z_*j*_ is centered around zero, the parameters of the QTL effects can be interpreted as follows: α_q^*^_ corresponds to the effect of QTL in the average environment (that is, when Z = 0); β_q^*^_ corresponds to the change of the QTL effect per unit of change of the covariable's value; and the random term a_jq^*^_ corresponds to the residual (unexplained) QTL effect, with a_jq^*^_ ~ N(0, σ^2^_aq^*^_). For example applying model 16 to QTL_1,141_, and with minimum temperature during flowering time as covariable, showed a significant reaction of QTL_1,141_ to changes in the minimum temperature during flowering, with β estimate equal to −0.040 ton ha^−1^ °C^−1^. We can interpret this result saying that when the maternal allele is replaced by the paternal allele, we expect a yield decrease of 0.040 ton ha^−1^ for each degree Celsius of increase in the minimum temperature during flowering.

The example assumed a simple linear relationship between the QTL effect and a single environmental covariable, but more complex explanatory models can be constructed. For example, it is possible to include higher order terms to model the response curve (e.g., a quadratic term), to use spline formulations, or to include more than one environmental covariable in the model. It is important to mention that a close interaction with physiologists is crucial to explore and select biologically sound models.

## Conclusion

We have discussed a suite of statistical models that are useful to plant breeding practitioners who are dealing with GEI. What all models have in common is that they make an attempt to replace the ANOVA GEI_*ij*_ term by product terms of genotypic parameters/covariates and environmental parameters/covariates, with as examples b_*i*_z_*j*_ (FW, AMMI, and GGE), b_*i*_Z_*j*_ (factorial regression), and X_*i*_α_*j*_ (QTL mapping). For some models no other information than the two-way table of means is required (FW, AMMI, and GGE), others require explicit environmental (factorial regression) and/or genotypic information (QTL models). For exploring patterns of GEI, FW, AMMI, and GGE are very useful. For prediction and understanding, factorial regression and QTL models are more appropriate.

### Conflict of interest statement

The authors declare that the research was conducted in the absence of any commercial or financial relationships that could be construed as a potential conflict of interest.
